# Antifungal Activity and Biocompatibility of α-AgVO_3_, α-Ag_2_WO_4_, and β-Ag_2_MoO_4_ Using a Three-Dimensional Coculture Model of the Oral Mucosa

**DOI:** 10.3389/fbioe.2022.826123

**Published:** 2022-02-14

**Authors:** Bruna Natália Alves da Silva Pimentel, Freddy Humberto Marin-Dett, Marcelo Assis, Paula Aboud Barbugli, Elson Longo, Carlos Eduardo Vergani

**Affiliations:** ^1^ Laboratory of Applied Microbiology, Department of Dental Materials and Prosthodontics, School of Dentistry, São Paulo State University (UNESP), Araraquara, Brazil; ^2^ Department of Clinical Analysis, School of Pharmaceutical Sciences, São Paulo State University (UNESP), Araraquara, Brazil; ^3^ CDMF, LIEC, Chemistry Department, Federal University of São Carlos (UFSCar), São Carlos, Brazil

**Keywords:** silver, microcrystals, infection, *Candida albicans*, 3D cultures, cytokines

## Abstract

Fungal infections have become a major concern in the medical community, especially those caused by *Candida* spp. Within this species, *Candida albicans* stands out for being an opportunistic commensal fungus that can cause superficial and invasive infections. Current antifungal therapy involves the local and/or systemic use of drugs such as azoles, polyenes, and echinocandins. These antifungals are based on highly specific target sites, and the development of resistance may occur with changes in the enzymatic pathways that serve as the drug targets. Thus, the development of new antifungal drugs is highly recommended to prevent drug resistance. The present investigation evaluated the antifungal activity of silver-containing microcrystals such as silver vanadate (α-AgVO_3_), silver tungstate (α-Ag_2_WO_4_), and silver molybdate (β-Ag_2_MoO_4_). In addition to having antimicrobial activity, such compounds should not cause damage to underlying tissues. Thus, to better assess the biocompatibility of new compounds, a new three-dimensional (3D) coculture model involving three cell lines was developed. The validation of the model was based on fluorescent markers and confocal laser microscopy. The biocompatibility of silver-containing microcrystals was evaluated by MTT (3-(4,5-dimethylthiazol-2-yl)-2,5-diphenyltetrazolium bromide) assay. 3D coculture was infected with *C. albicans* biofilm and challenged with α-AgVO_3_, α-Ag_2_WO_4_, and β-Ag_2_MoO_4_. The action of microcrystals on *C. albicans* biofilm was evaluated by colony-forming units (CFU/ml) and LIVE/DEAD staining. In addition, production of proinflammatory cytokines interleukin 6 (IL-6), IL-8, IL-1β, and tumor necrosis factor α (TNF-α) was measured by cytometric bead array kit using flow cytometry. The 3D coculture model described here proved to be adequate to assess both the biocompatibility of the new materials and the infectious processes. Regarding the biocompatibility of the microcrystals, only α-AgVO_3_ (15.62 µg/ml) showed a decrease in cell viability. The antibiofilm activity of α-Ag_2_WO_4_ was similar to that of the standard drug (fluconazole). Although α-Ag_2_WO_4_ was able to induce the production of IL-6, IL-8, and IL-1β, no differences in cytokine production were observed between noninfected and infected models treated with this microcrystal. β-Ag_2_MoO_4_ inhibits the production of TNF-α in the infected model; however, it showed no antibiofilm activity. Based on the biocompatibility and antifungal findings, α-Ag_2_WO_4_ is a promising material for treating *C. albicans* infection.

## Introduction

Opportunistic fungal infections have been described as important causes of morbidity and mortality (Naglik et al., 2017). Among several opportunistic fungi, *Candida albicans* is considered one of the most relevant, as it is responsible for a large number of fungal infections (Pellon et al., 2020). In patients with systemic fungal infections, there is a predominance of *C. albicans* in clinical isolates (58%), with the lungs being a frequent site of colonization (Xie et al., 2008; Pellon et al., 2020). The presence of *C. albicans* associated with systemic and/or local factors, such as chronic diseases, immunosuppression, dry mouth, and poor oral hygiene (Salerno et al., 2011), is related to higher rates of morbidity and mortality in these patients (Sudbery et al., 2004; Salerno et al., 2011).

The pathophysiology of *C. albicans* is closely influenced by several virulence factors such as the capacity to adhere to host tissues, the secretion of hydrolytic enzymes (proteases, phospholipases, and hemolysins), and the ability to form biofilm, a highly organized structure that protects the microorganism from environmental changes, including antifungal agents (Sardi et al., 2013). Currently, local and/or systemic antifungal therapy includes the use of several drugs to treat *C. albicans* infections, with echinocandins, polyenes, and azoles being the most commonly used. These drugs act by preventing the synthesis or binding to components of the cell membrane of fungi, such as ergosterol, thus causing their death (Andriole, 2000). Despite their relatively good performance and clinical success, the prolonged use of these antifungals can cause kidney and liver damage (Andriole, 2000). In addition, the indiscriminate use of these drugs may lead to increased tolerance/resistance of microorganisms due to molecular mechanisms of protection, such as the decrease in the permeability of the membrane to the drug (Canuto and Rodero, 2002). Thus, identifying novel multitarget antifungal agents to overcome these limitations represents an urgent need in the field.

New materials that physically and/or chemically inactivate microorganisms have been widely studied, mainly because they have a low propensity to cause microbial resistance (Kim et al., 2009; Panáček et al., 2009; Lipovsky et al., 2011; Junqueira et al., 2012; Dovigo et al., 2013; Longo et al., 2014; Fabbro et al., 2016; de Oliveira et al., 2017; Foggi et al., 2017a; Foggi et al., 2017b; Hamida et al., 2020). Among these materials, the silver-containing microcrystals silver vanadate (α-AgVO_3_), silver tungstate (α-Ag_2_WO_4_), and silver molybdate (β-Ag_2_MoO_4_) have been shown to promote growth inhibition and/or death of microorganisms such as *C. albicans* and methicillin-resistant *Staphylococcus aureus* in their free (planktonic) form (Longo et al., 2014; Fabbro et al., 2016; de Oliveira et al., 2017; Foggi et al., 2017a; Foggi et al., 2017b; Assis et al., 2018; Assis et al., 2019; Pimentel et al., 2020). The proposed mechanism of action is based on theoretical calculations where, through the generation of free radicals such as hydroxyl (OH*), superoxide (O′_2_), and hydroperoxyl (HO_2_*), important components of microorganisms such as polysaccharides, lipids, and cell membrane proteins are affected, thus altering their integrity and causing microorganism death (Longo et al., 2014; Fabbro et al., 2016; de Oliveira et al., 2017; Foggi et al., 2017a; Foggi et al., 2017b). Despite these favorable results, little is known about the action of these microcrystals on microbial biofilms.

An important concern regarding such new materials is their biocompatibility with tissues, as the free radicals generated can also affect mammalian cells (Schieber and Chandel, 2014). Although reactive oxygen species (ROS) act as molecular signals in the regulation of physiological processes, they can lead to hyperactivation of the inflammatory response at high levels (Schieber and Chandel, 2014). Oxidative stress generated by ROS causes the activation of the MAPK (mitogen-activated protein kinase) signaling pathway, which induces transcription factors such as NF-κB (nuclear factor κB), leading to the production of proinflammatory cytokines (Park and Park, 2009). Previous studies based on monolayer cell growth model have shown that, at some concentrations, α-AgVO_3_, α-Ag_2_WO_4_, and β-Ag_2_MoO_4_ microcrystals were not cytotoxic (Chávez et al., 2018; Assis et al., 2019; Pimentel et al., 2020). Although traditional monolayer cell culture models are still predominant, they may fail to reproduce the complex and dynamic environment of *in vivo* tissues. As a result, there may be false results or inconsistencies, as in such model the cells are “forced” to adapt to a rigid and artificial surface (Brohem et al., 2010). Another important point is regarding the type of cells in the culture, as it is known that the greater the number of cell lines involved, the more reliable the responses obtained in *in vitro* assays (Zhou et al., 2015).

In the present investigation, a three-dimensional (3D) cell coculture model containing fibroblasts, keratinocytes, and monocytes was developed to assess the biocompatibility and inflammatory responses of α-AgVO_3_, α-Ag_2_WO_4_, and β-Ag_2_MoO_4_ microcrystals, as well as to evaluate the antifungal activity of the materials against *C. albicans* biofilms.

## Materials and Methods

### Microcrystal’s Synthesis and Characterization

The synthesis of α-AgVO_3_, α-Ag_2_WO_4_, and β-Ag_2_MoO_4_ microcrystals was performed using the coprecipitation method in an aqueous medium, as published in previous studies (Fabbro et al., 2016; Foggi et al., 2017a; Pimentel et al., 2020). First, 1 × 10^−3^ mol of the Ag^+^ precursor (AgNO_3_, Cennabras, 99.98%) was diluted in 50 ml of distilled water. Concomitantly, the precursors of the lattice formers were diluted in stoichiometric amounts in 50 ml of distilled water [1 × 10^−3^ mol of NH_4_VO_3_ (Aldrich, 99.99%), or 5 × 10^−4^ mol of Na_2_WO_4_.·2H_2_O (Aldrich, 99.99%), or Na_2_MoO_4_ (Alpha Aesar, 99.98%)]. Temperatures of 10°C for α-AgVO_3_ and 70°C for α-Ag_2_WO_4_ and β-Ag_2_MoO_4_ were used. After the solutions reached the required temperatures, the lattice former solutions were added to the Ag^+^ solution, instantly forming the precipitates. These precipitates were washed with distilled water to pH 7 and oven-dried at 60°C for 12 h. After synthesis, all three microcrystals were diluted in phosphate-buffered saline solution (PBS) at a stock concentration of 2 mg/ml. The materials were characterized by X-ray diffraction (XRD) using a Rigaku-DMax/2500PC (Rigaku, Tokyo, Japan) with Cu Kα radiation and by micro-Raman spectroscopy using a Horiba Jobin-Yvon spectrophotometer coupled to a CCD Synapse detector and using a 514-nm argon laser.

The microcrystals concentrations evaluated in the present study were based on previous studies (Fabbro et al., 2016; Foggi et al., 2017a; Pimentel et al., 2020) according to the minimal inhibition concentration and minimal fungicidal concentration against *C. albicans* (Table 1).

**TABLE 1:** Microcrystals and their respective minimal inhibition concentration (MIC) and minimal fungicidal concentration (MFC) according to previous studies.

Microcrystal	Concentration (µg/ml)
α-AgVO_3_	3.9 (MIC) (Pimentel et al., 2020)
	15.62 (MFC) (Pimentel et al., 2020)
α-Ag_2_WO_4_	7.81 (MIC/MFC) (Foggi et al., 2017a)
β-Ag_2_MoO_4_	15.62 (MIC/MFC) (Fabbro et al., 2016)

### Cell Culture Conditions

For the present study, three cell lines were used: FGH (Rio de Janeiro Cell Bank; code: 0089), THP-1 (Rio de Janeiro Cell Bank; code: 0234), and NOK-si cells (kindly provided by Professor Carlos Rossa Jr., from the Cellular and Molecular Biology Laboratory, Department of Periodontics, School of Dentistry, São Paulo State University—UNESP). NOK-si and FGH cells were cultured in high-glucose (4.5 g/L) Dulbecco eagle modified medium (DMEM; Gibco, NY, USA) supplemented with l-glutamine (2 mM/L; Lonza, Basel, Switzerland), fetal bovine serum (FBS; 10% vol/vol; Gibco), and 1% vol/vol antibiotic/antimycotic solution (penicillin G—10.000 µg/ml, streptomycin—10.000 µg/ml and amphotericin B—25 µg/ml) (Sigma-Aldrich, MO, USA) (Castilho et al., 2010). THP-1 cells were cultured in Roswell Park Memorial Institute medium (RPMI-1640) supplemented with 2 mM glutamine, 10 mM HEPES (Sigma-Aldrich), 1 mM sodium pyruvate, 4.5 g/L glucose, 1.5 g/L sodium bicarbonate, 10% vol/vol FBS, and 0.09% vol/vol β-mercaptoethanol (Gibco). All cells were incubated at 37°C with 5% CO_2_.

### Three-Dimensional Cell Coculture Model

For the development of the 3D cell coculture model, cells were grown until they reached 90% confluence. The cells were washed with sterile PBS (pH 7.2), detached with trypsin solution (0.05% vol/vol)/EDTA (0.53 mM/L) (Sigma-Aldrich), and centrifuged at 400 × *g* for 5 min. The protocol used was adapted from Haro Chávez et al. (2018). Briefly, 2.3 ml of DMEM medium, 450 µl of FBS, 1.5 ml of rat tail collagen (First Link, Wolverhampton, United Kingdom), 200 µl of NaOH (1 M), 250 µl of FGH cell suspension (6 × 10^6^ cells/ml), and 100 µl THP-1 cell suspension (1.5 × 10^6^ cells/ml) were added to a 50-ml conical tube. The solution was carefully mixed and plated in a 24-well plate. Next, 500 µl of the solution was added to each well, and the plate was incubated until polymerization of the collagen at 37°C with 5% CO_2_. After polymerization, 75 µl of NOK-si cell suspension (2 × 10^6^ cells/ml) was added. The plate was incubated for 24 h for adhesion and formation of the keratinocyte layer.

### Confocal Laser Scanning Microscopy of the Noninfected 3D Coculture Model

To characterize the 3D coculture model, confocal laser scanning microscopy (CLSM) assays were performed. All CLSM assays were performed with the LSM 800 Carl Zeiss with ZenBlue Software 2.3. Prior to making the 3D coculture model, THP-1 cells were stained with Cell Trace CSFE probe (Thermo Fisher Scientific, MA, USA) to confirm this cell phenotype in the deep layers of the 3D cocultures. So, THP-1 cells were washed twice with PBS and centrifuged at 400 × *g* for 5 min before their collection. The pellet was resuspended and diluted in PBS to obtain a 1.5 × 10^6^ cells/ml THP-1 cell suspension. Next, 2 µl of the Cell Trace CFSE loading solution was added to the cell suspension, and the tube was incubated at 37°C. After 20 min, 20 µl of FBS was added, and the cells were centrifuged at 400 × *g* for 3 min. The pellet was washed twice with PBS supplemented with 2% vol/vol FBS. After the washing steps, the pellets were resuspended in 1 ml of DMEM media without FBS prior to use for 3D culture formation (already described in *Cell Culture Conditions* section). For CLSM, 3D cocultures were washed with PBS and fixed with paraformaldehyde (4% vol/vol) for 30 min at 37°C. Next, the plates were washed with PBS, and Triton (0.1% vol/vol) was added. After 10 min of incubation, the wells were washed twice with PBS and labeled with ActinRed™ 555 ReadyProbes™ reagent (Thermo Fisher Scientific) for 30 min. Then, 3D cocultures were washed with PBS before CLSM visualization. For the image acquisition, 488- and 561-nm lasers were used.

For the 3D cocultures exposed to α-AgVO_3_, α-AgWO_4_, and β-AgMoO_4_, the media was collected and stored at −20°C for cytokine analysis, previously to the staining protocol. Next, 500 µl of the Hoechst 33342 and propidium iodide (PI) (1:1,000) (Sigma-Aldrich) were added to each well and incubated for 10 min, protected from light. Next, the 3D cocultures were washed with PBS; 300 µl of paraformaldehyde (4% vol/vol) were added to each well, and the plates were incubated for 20 min at 37°C. After 20 min, the plates were washed with PBS and 300 µl of Triton (0.1% vol/vol) were added and incubated for 10 min. Next, the wells were washed with PBS. After washing, Alexa Fluor® 488 Phalloidin (Thermo Fisher Scientific) was added (300 µl/3D coculture model) and incubated for 30 min. Next, 3D cocultures were washed, and finally, 500 µl of PBS was added prior to the CLSM assays. For the image acquisition, 405- and 488-nm lasers were used in the *X*, *Y*, and *Z* axes.

### Cytotoxic Assay in Noninfected 3D Coculture Model

For the assessment of the α-AgVO_3_, α-AgWO_4_, and β-AgMoO_4_ cytotoxicity, the microcrystals were added to the 3D coculture model and cocultured for 24 h. The cell viability was determined by MTT (3-(4,5-dimethylthiazol-2-yl)-2,5-diphenyltetrazolium bromide) by tetrazolium salt (Sigma-Aldrich) assay, as described by Dias et al. (2017). Briefly, the media was collected from the plates, and the wells were washed with PBS. Next, 400 µl of MTT in RPMI without phenol was added (2 mg/ml) in each well, and the plate was incubated for 4 h at 37°C and 5% CO_2_. After solubilization of formazan crystals, the plate was measured at 562 nm in an EZ Read 400 Microplate reader (BioChrom) with assistance from the Adapt 2.0 BioChrom software. The assay was performed in triplicate on three different occasions.

### Three-Dimensional Coculture Model Infection With *C. albicans* Biofilm

*C. albicans* is the most prevalent fungus related to oral candidiasis, a recurrent oral infection in users of removable dentures, as well as in patients admitted to ICUs (Sardi et al., 2013). Thus, this work focused on evaluating the infection of a *C. albicans* strain in the 3D coculture model. For the infection model, a *C. albicans* strain (ATCC 90028) was used. The yeast was unfrozen and cultured in a Sabouraud dextrose agar (SDA; KASVI, Parana, Brazil) plate supplemented with chloramphenicol (0.1 g/L) and incubated for 24 h at 37°C. For the preinoculum, five colonies were cultured in 10 ml of yeast nitrogen base (YNB; BD Difco, CA, USA) supplemented with 100 mM glucose at 37°C. After 16 h, the culture was diluted 1:20 in a fresh YNB medium and incubated at 37°C for 8 h for inoculum formation. Next, the yeast was washed twice with PBS and centrifuged at 250 × *g* for 10 min at 4°C. The pellet was resuspended in RPMI-1640 buffered with HEPES (25 mM) and supplemented with 2 mM of l-glutamine and sodium bicarbonate (2 g/L). Then, to obtain a *C. albicans* cell suspension of 3 × 10^4^ colony-forming unit (CFU)/ml, a dilution was performed. Finally, 100 μl of the *C. albicans* cell suspension was added to each well of the 24-well plate containing the 3D coculture model. The plates were incubated for 24 h at 37°C with 5% CO_2_.

### Evaluation of the Antifungal Effect of the Silver-Containing Microcrystals

To evaluate the antifungal effect of the silver-containing microcrystals, CFU/ml by plate count was performed. For this assay, the 3D coculture model was collected and vortexed with 1 ml of PBS for 5 min. After 5 min, 100 µl was collected and diluted 1:1,000 in PBS. Then, 20 µl of each diluted solution was plated in SDA plates and incubated at 37°C for 24 h. Next, the number of colonies was counted, and the CFU/ml was calculated. Fluconazole (FCZ) at 120 µg/ml was used as standard drug control. The experiment was performed in triplicate on three different occasions.

The antifungal effect of silver-containing microcrystals on 3D coculture was also evaluated by CLSM using LIVE/DEAD™ BacLight™ Bacterial Viability Kit (Molecular Probes, OR, USA). Briefly, after collecting and vortexing the 3D coculture model as described previously, an aliquot of 100 µl of each experimental group was stained with PI and Syto 9 (1:1,000) diluted in PBS. Then, 1 ml of the solution was added to confocal plates and incubated for 30 min protected from light, followed by CLSM observation. The laser was fixed at 488 nm with 4% power for the excitation of both probes. For emission, 530 nm was used for Syto9 detection and 610 nm for PI detection, both with a gain of 700 V in the *X*, *Y*, and *Z* axes. FCZ at 120 µg/ml was used as standard drug control. The experiment was performed in duplicate on two different occasions.

### Confocal Laser Scanning Microscopy of the Infected 3D Coculture Model

To characterize the 3D coculture model, CLSM assays were performed. First, THP-1 cells were stained with a Cell Trace CSFE probe as already described (*Three-Dimensional Cell Coculture Model*). After ActinRed™ staining, CalcoFluor White (CW) probe (Sigma-Aldrich) was added. For this, 20 μl of CW and 20 μl of 10% vol/vol KOH were added to each well, and the plate was incubated at room temperature. After 10 min, the wells were washed with PBS prior to the CLSM assay. For the image acquisition, 405- and 561-nm lasers were used.

For the infected 3D coculture model challenged with α-AgVO_3_, α-AgWO_4_, and β-AgMoO_4_, the media were collected and stored at −20°C for cytokine analysis, previously to staining protocol. Next, 500 µl of PI (1:1,000) was added to each well and incubated for 10 min, protected from light. After 10 min, the 3D cocultures were washed with PBS. Next, 300 µl of paraformaldehyde (4% vol/vol) was added to each well, and the cultures were incubated for 20 min at 37°C. The plates were washed with PBS and 300 µl of Triton (0.1% vol/vol) was added and incubated for 10 min. Then, the wells were washed with PBS. Thereafter, Alexa Fluor® 488 Phalloidin (Thermo Fisher Scientific) was added and incubated for 30 min. The 3D cocultures were washed, and finally, 500 µl of PBS was added prior to the CLSM assays. For the image acquisition, 405- and 488-nm lasers were used in the *X*, *Y*, and *Z* axes.

### Cytometric Bead Array

For cytokine detection, the BD™ cytometric bead assay was used. Briefly, the lyophilized Human Inflammatory Cytokines Standards were reconstituted with 2 ml of Assay Diluent. Next, a serial dilution was performed until 1:256 dilution. Negative control was prepared with only Assay Diluent. Then, a mix of Capture Beads was prepared. In cytometry, 50 µl of the mixed Capture Bead was added to tubes properly named with standard curve points and samples. Next, 50 μl of the Human Inflammatory Cytokines Standard previously serially diluted was added to the standard curve tubes, and 50 μl of each sample was added to its respective tube. Then, 50 μl of the Human Inflammatory Cytokines PE Detection Reagent was added to all the tubes, which were incubated at room temperature and protected from light. After 3 h, 1 ml of wash buffer was added to each tube followed by centrifugation at 200 × *g* for 5 min. The supernatant was then discarded and 300 μl of wash buffer was added to each tube. The samples were acquired in a BD FACSAria™ Fusion Flow Cytometer. Data were analyzed with the FCAP Array v. 3.1 software.

### Statistical Analysis

To assess the distribution and homoscedasticity of the data, the Shapiro–Wilk and Levene tests were applied. As the data of CFU/ml did not show normal distribution, the analysis was performed using the Kruskal–Wallis test followed by Dunn *post hoc* test. The data obtained from the MTT and cytometric bead array (CBA) tests presented normal distribution and equality of variances. Thus, the one-way analysis of variance test was performed, followed by Tukey *post hoc* test for MTT data. CBA data were evaluated by Student t-test. All data were evaluated using the GraphPad Prism software version 5.0 (GraphPad Software, CA, USA), where *p* < 0.05 was considered statistically significant.

## Results

### Microcrystal Characterization

**FIGURE 1:**
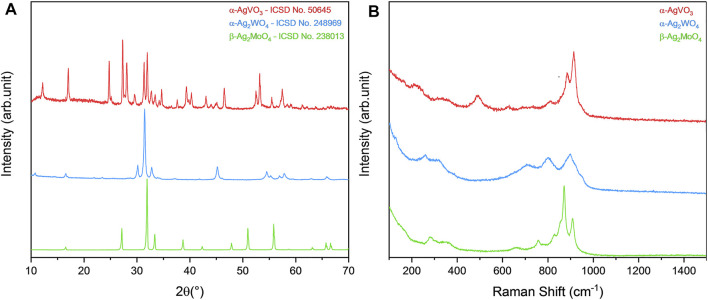
Characterization of α-AgVO_3_, α-Ag_2_WO_4_, and β-Ag_2_MoO_4_ microcrystals. **(A)** XRD pattern and **(B)** micro-Raman spectroscopy.

After synthesis, all materials were structurally characterized in order to observe their respective crystalline phases, as well as their purity. By both XRD and micro-Raman spectroscopy (Figure 1), it was possible to identify the formation of the monoclinic phase in α-AgVO_3_, characteristic of such compound, with space group *C2/c*, in agreement with the Inorganic Crystal Structure Database (ICSD), card no. 50645 (de Oliveira et al., 2017). For α-Ag_2_WO_4_, the monoclinic structure with *Pn2n* space group was obtained, in accordance with ICSD card no. 248969 (Assis et al., 2018; Assis et al., 2019; Assis et al., 2020; Assis et al., 2021). The β-Ag_2_MoO_4_ was related to ICSD card no. 238013, with cubic structure and *Fd3m* space group (Foggi et al., 2020; Penha et al., 2020). In all cases, no additional phases were observed, indicating the high purity of the materials.

### Characterization of the 3D Coculture Model

**FIGURE 2:**
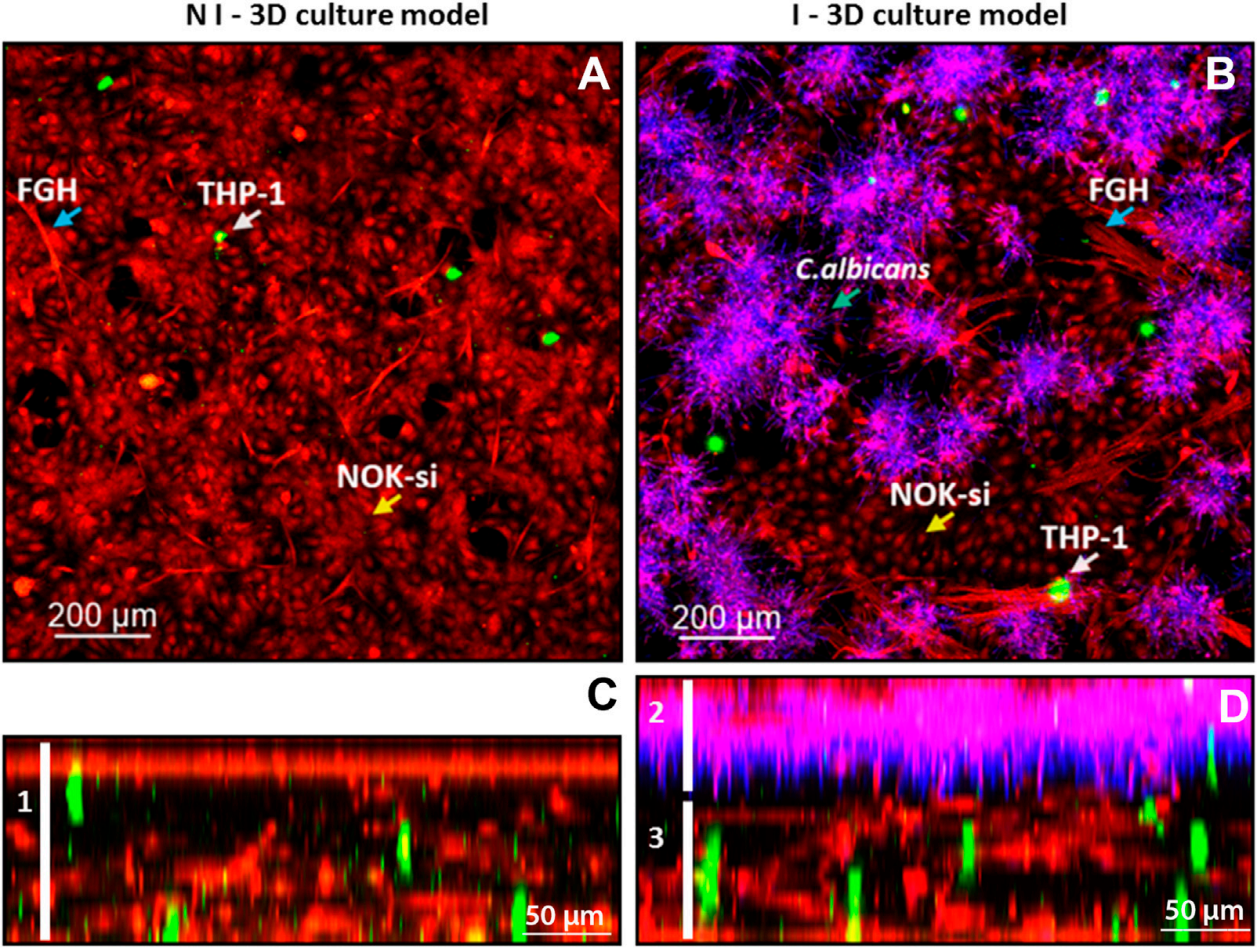
Confocal fluorescence microscopy of the infected and noninfected 3D coculture models, collagen matrix stained with ActinRed 555 (red, cytoskeleton), CSFE (green, inflammatory cells), and CalcoFluor White (purple, *Candida albicans*). **(A)** Upper view of the noninfected 3D coculture model: blue arrow, FGH cells; white arrow, THP-1 cells; and yellow arrow, NOK-si cells. **(B)** Upper view of the infected 3D coculture model: blue arrow, FGH cells; white arrow, THP-1 cells; and yellow arrow, NOK-si cells; blue arrow over the CalcoFluor White staining, *C. albicans*. **(C)** Side view of the noninfected 3D coculture model, demonstrating the formation of a monolayer of keratinocyte and the presence of the inflammatory cells within the 3D coculture model. **(D)** Side view of the infected 3D coculture model, demonstrating a formation of *C. albicans* biofilm adhered to the keratinocyte monolayer.

To characterize the 3D coculture model, a CLSM analyses was performed. It was observed that the noninfected 3D coculture was formed by an epithelial layer of 70-µm thickness connected to the collagen matrix layer of 130-µm thickness, containing FGH (red) and THP-1 cells (green) (Figure 2, left field, white bar 1). The 3D coculture model infected with *C. albicans* biofilm had a total thickness of 210 µm, with an epithelial layer of 80 µm colonized by the biofilm (Figure 2, right field, bar 2) and connected to the collagen matrix layer containing inflammatory cells (green) of 130 µm (Figure 2, right field, line 3).

### Biocompatibility of the Microcrystals in Noninfected 3D Coculture Model

**FIGURE 3:**
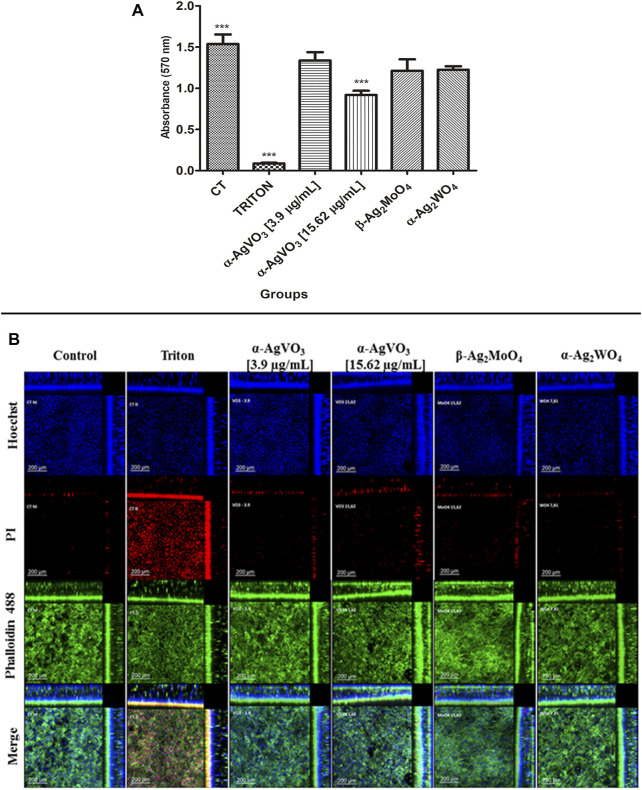
Viability assay and confocal imaging of the noninfected 3D coculture exposed to the compounds. **(A)** Viability assay measured with MTT assay; a statistical difference was observed in α-AgVO_3_ (15.62 µg/ml) and the positive control; ***p < 0.0001. **(B)** Confocal imaging of the noninfected 3D coculture, Hoechst staining for nuclei, propidium iodide (PI) staining for death cells, Alexa Fluor® 488 Phalloidin staining for cytoskeleton, and merge of the microscopy images of all the probes.

To assess biocompatibility, the 3D coculture models were challenged with the microcrystals for 24 h and then analyzed by MTT and CLSM (Figure 3). The microcrystals α-Ag_2_WO_4_, α-AgVO_3_ (3.9 µg/ml), and β-Ag_2_MoO_4_ showed no decrease in cell viability as compared with control (Figure 3A). However, a significant decrease in cell viability (*p* < 0.0001) was observed when the 3D coculture model was challenged with α-AgVO_3_ at 15.62 µg/ml (Figure 3A). Data obtained by MTT were confirmed by CLSM (Figure 3B), where different degrees of cell death in 3D coculture models were detected by positive labeling with PI (Figure 3B, red panel).

### Antibiofilm Effect of the Silver-Containing Microcrystals

**FIGURE 4:**
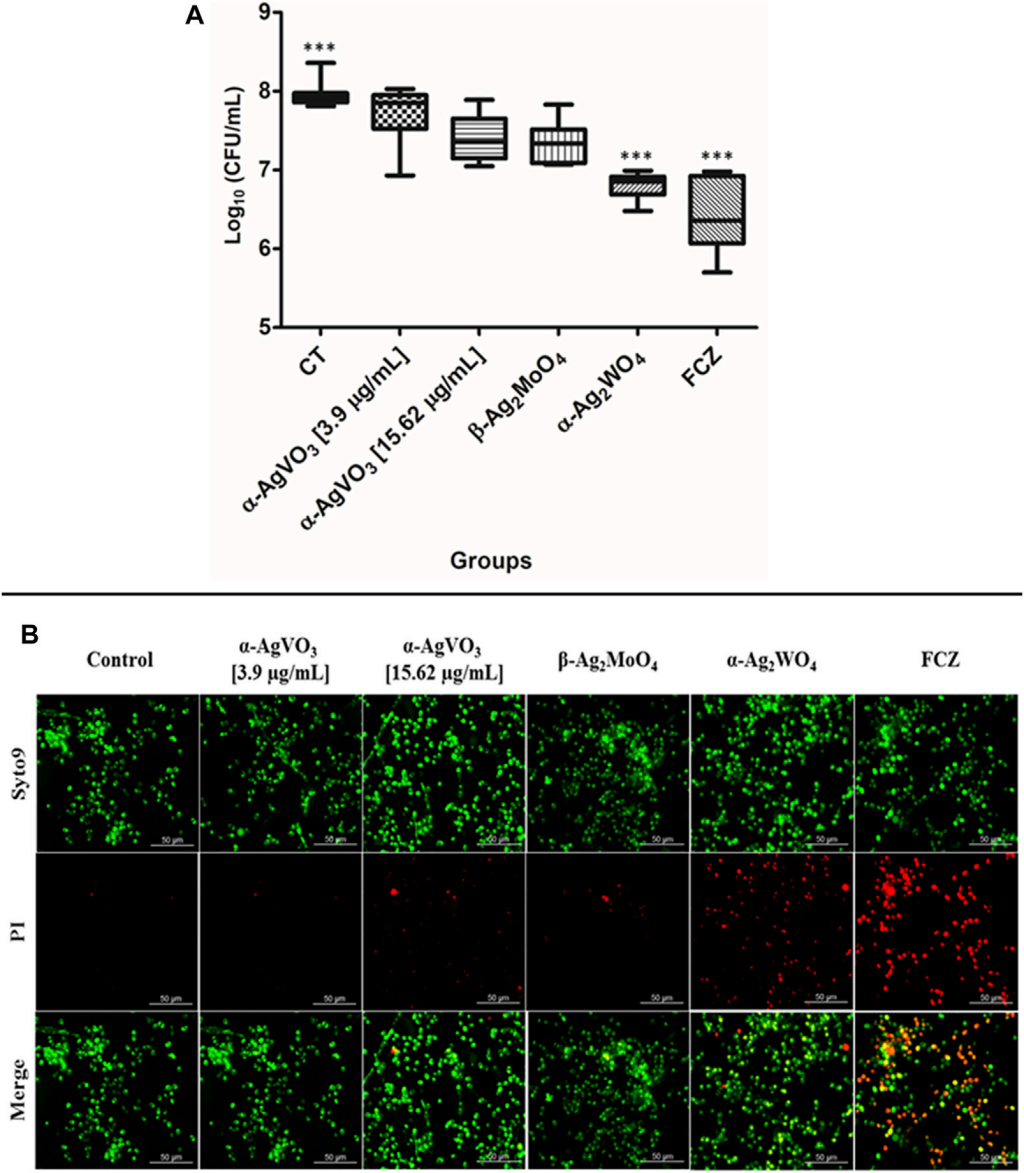
Antifungal effects of the microcrystals against *C. albicans–*infected 3D coculture models. **(A)** Logarithmic CFU/ml counts of *C. albicans* after the exposition to the microcrystals in the infected 3D cocultured model. Statistical difference observed in FCZ and α-AgWO_4_; ***p < 0.0001. **(B)** LIVE/DEAD confocal imaging of *C. albicans* cells exposed to the microcrystals, Syto9 staining for the live *C. albicans* cells, propidium iodide (PI) for the *C. albicans* dead cells, and the merge of the images.

To evaluate the antibiofilm activity of microcrystals α-AgVO_3_, α-Ag_2_WO_4_, and β-Ag_2_MoO_4_, CFU/ml was performed (Figure 4A). It was not possible to observe significant reductions in microbial load when *C. albicans* biofilms were challenged with α-AgVO_3_ (3.9 and 15.62 µg/ml) and β-Ag_2_MoO_4_ microcrystals. On the other hand, α-Ag_2_WO_4_ showed a significant reduction (*p* < 0.0001) of approximately 1.5 log_10_, with no significant difference to the standard treatment with FCZ. These findings were confirmed by LIVE/DEAD staining (Figure 4B), where it was possible to detect *C. albicans* cells labeled with PI (red) only for those 3D cocultures challenged with α-Ag_2_WO_4_ and FCZ (Figure 4B; red cells).

**FIGURE 5:**
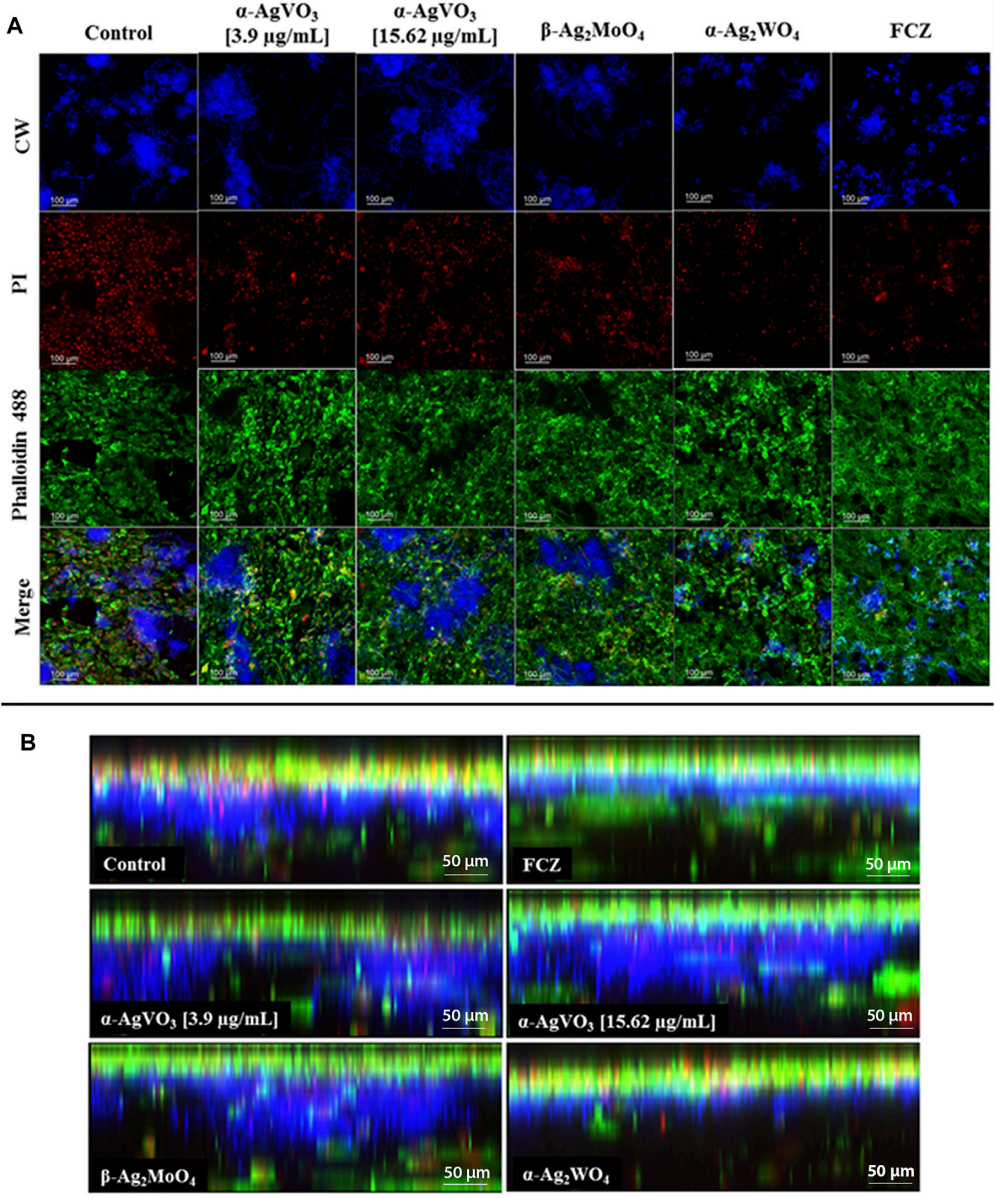
Confocal microscopy of the infected 3D coculture models. CalcoFluor White staining for *C. albicans* (blue), propidium iodide (PI) staining for death cells (red), Alexa Fluor® 488 Phalloidin staining for cytoskeleton (green), and the merge of the images. **(A)** Upper view of the confocal microscopy of the infected 3D coculture models exposed to the microcrystals. **(B)** Side view of the confocal microscopy of the infected 3D coculture models exposed to the microcrystals.

CLSM of the 3D coculture models confirmed the findings of CFU/ml, where α-Ag_2_WO_4_ and FCZ showed similar antibiofilm activity (Figure 5A). In these two groups, positive staining for CW (Figure 5A, blue panel) was significantly lower than the infected control, α-AgVO_3_ (3.9 and 15.62 µg/ml) and β-Ag_2_MoO_4_ groups. Further, a lower number of epithelial dead cells labeled with PI were seen in α-Ag_2_WO_4_ and FCZ groups as compared with the other groups (Figure 5A, red panel). Similarly, α-Ag_2_WO_4_ and FCZ were less harmful to epithelial layer as revealed by Alexa Fluor® 488 Phalloidin stain (Figure 5A, green panel). Figure 5B also shows better preservation of the epithelial layer when 3D coculture models were challenged with α-Ag_2_WO_4_ and FCZ. In these groups, the penetration of *C. albicans* (blue fluorescence of CW) was restricted to the epithelial layer (green fluorescence of Alexa Fluor® 488 Phalloidin in the upper layer of the 3D culture). In contrast, in the infected control group and α-AgVO_3_ and β-Ag_2_MoO_4_ experimental groups, it was possible to notice the presence of black niches, representing the absence of Alexa Fluor® 488 Phalloidin labeling, and the increased blue fluorescence niches with CW, which is related to higher invasiveness of the *C. albicans* biofilm into the epithelial layer of the 3D cultures (Figure 5B, blue, green and merge panels).

### Cytokine Production

**FIGURE 6:**
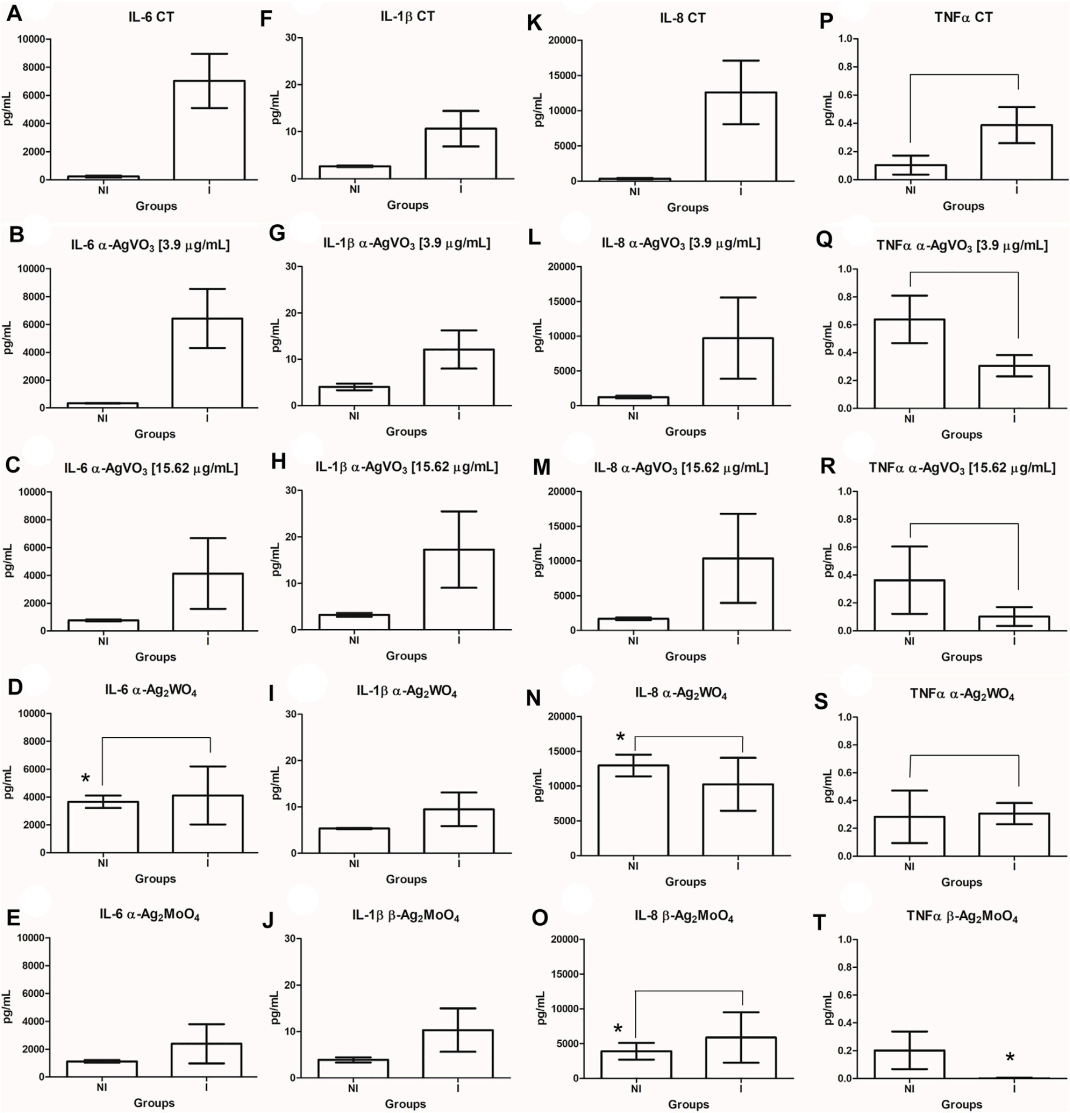
Comparisons in the cytokine (IL-6, IL-1β, IL-8, and TNF-α) production between the infected and noninfected 3D co-culture models exposed to the microcrystals. **(A-E)**: IL-6; **(F-J)**: IL-1β; **(K-O)**: IL-8; **(P-T)**: TNFα. *denotes statistical difference between each experimental group and its respective control. Connecting bars denote no significant difference between groups.

The cytokine production by 3D coculture models was assessed by flow cytometry (Figure 6). In noninfected 3D coculture models challenged with microcrystals, α-Ag_2_WO_4_ promoted a significant increase in interleukin 6 (IL-6) production compared with control (*p* = 0.0414; Figure 6D). A significant increase in IL-8 production was also observed when the models were challenged with α-Ag_2_WO_4_ (*p* = 0.0053; Figure 6N) and β-Ag_2_MoO_4_ (*p* = 0.0090; Figure 6O). When compared with noninfected control, no statistically significant differences in the production of the cytokines [IL-6, IL-1β, IL-8, and tumor necrosis factor α (TNF-α)] were found, when the 3D coculture models were challenged with α-AgVO_3_ (3.9 and 15.62 µg/ml). In 3D coculture models infected with *C. albicans* biofilm (infected control), a significant increase in the production of IL-6 (*p* = 0.0023; Figure 6A), IL-1β (*p* = 0.0036; Figure 6F) and IL-8 was observed (*p* = 0.0006; Figure 6K) as compared with the noninfected control. However, no significant increase in TNF-α was observed (*p* = 0.4351; Figure 6P). In general, after the infected 3D coculture models were challenged with microcrystals, no significant differences in the production of the cytokines were observed in relation to noninfected 3D coculture models also challenged with microcrystals. The only exception was the inhibition of TNF-α production in infected models challenged with β-Ag_2_MoO_4_ (*p* = 0.0012; Figure 6T).

## Discussion

In the present study, a 3D cell coculture model was developed and used to evaluate the biocompatibility and antibiofilm activity of silver-containing microcrystals. Several 3D cell coculture models are currently used to study infectious processes, cell behavior, neoplasms, and biocompatibility (Mostefaoui et al., 2002; Klausner et al., 2007; Moharamzadeh et al., 2008; Kinikoglu et al., 2009; Yadev et al., 2011; Pinnock et al., 2014; Heller et al., 2016; Dias et al., 2018). These 3D coculture models take into account the tissue anatomy to which they refer, making possible to better simulate a clinical situation when compared with monolayer cell cultures (Moharamzadeh et al., 2007). The complexity of these 3D coculture models should also be considered, as the greater the number of cell lines involved in making these cultures, the more reliable the responses obtained in *in vitro* assays. Thus, three cell lines were used in the present work. The proposed 3D coculture model included inflammatory cells (THP-1), embedded in a type 1 collagen matrix containing gingival fibroblasts (FGH). On the top surface of the polymerized matrix, it is possible to observe a layer of epithelial cells from the oral cavity (NOK-si). We also assessed the infectious process by *C. albicans* biofilms. 3D coculture model invasion by *C. albicans* cells was observed, which is in agreement with the literature, in both *in vivo* assays and *in vitro* models (Villar et al., 2007; Sun et al., 2010; Yang et al., 2014; Dias et al., 2018). Thus, the 3D coculture model described here was considered appropriate to assess the biocompatibility of the silver-containing microcrystals, the infectious process caused by *C. albicans* biofilm, and the antibiofilm activity of α-AgVO_3_, α-Ag_2_WO_4_, and β-Ag_2_MoO_4_ microcrystals.

The biocompatibility of the microcrystals on the 3D coculture model assessed by MTT and CLSM showed that only α-AgVO_3_ at 15.62 µg/ml was able to decrease cell viability in the 3D coculture model. Previous studies have already evaluated the cytotoxicity of α-AgVO_3_, β-Ag_2_MoO_4_, and α-Ag_2_WO_4_ microcrystals in monolayer cell culture, using FGH or NOK-si cell lines. In agreement to our results, these previous quantitative and qualitative assessments demonstrated that α-Ag_2_WO_4_ did not affect proliferation (Assis et al., 2019) and the mitochondrial enzymatic activity of FGH cells cultured in a monolayer or the cell viability within 3D collagen matrices (Chávez et al., 2018). Contrasting results from the present investigation have also been reported. Cytotoxic and genotoxic outcomes revealed that α-AgVO_3_ at 15.62 µg/ml did not affect NOK-si cell morphology, proliferation, or DNA integrity (Pimentel et al., 2020). Also, β-Ag_2_MoO_4_ was found to be cytotoxic to FGH cells (Chávez et al., 2018), as opposed to what was observed in the present work. These previous findings resulted from a single cell line and 2D culture model. Although they bring us important information, in a quickly and less costly way, they fail to reproduce the cell complex microenvironment (Brohem et al., 2010). When cells grow in 3D models, they have different proliferative capacity, metabolic functions, and responses to harmful agents then when they grow in 2D, due to cell–cell signaling (Brohem et al., 2010). Also, the use of more than one cell line enables a more reliable analysis of the cellular response, as in cellular coculture it is possible to more accurately simulate the cellular environment, especially when evaluating complex signals, such as the inflammatory responses (Zhou et al., 2015). This emphasizes the importance of more complex culture models, such as 3D coculture models, when assessing the biocompatibility of biomaterials (Klausner et al., 2007).

The antibiofilm activity was observed only in those infected 3D coculture models challenged with α-Ag_2_WO_4_, which was similar to standard control (FCZ). Previous studies have already shown that such microcrystals have activity against *C. albicans* cells in their free form (planktonic), but not on biofilm (Fabbro et al., 2016; Foggi et al., 2017a; Foggi et al., 2017b; Assis et al., 2019; Pimentel et al., 2020). The clinical importance of biofilms is widely known, especially in the oral cavity. The literature has shown that, during infectious processes, microorganisms are not found in their planktonic form, but rather form multicellular aggregates in host tissues (Soll, 2008). When organized in biofilms, the microorganisms are more tolerant to antimicrobial agents, so that the concentration of drugs needed to eliminate the microorganism is greater when compared with that needed to eliminate its planktonic form (Silva et al., 2017). Thus, it is essential to evaluate the antibiofilm properties when assessing new therapeutic strategies. In some studies showing the activity of silver nanoparticles against *C. albicans* biofilms (Martinez-Gutierrez et al., 2013; Monteiro et al., 2014; Lara et al., 2015), the concentrations needed to decrease the viability of *C. albicans* by at least 1 Log_10_ varied from 0.49 µg/ml (Lara et al., 2015) up to 1,000 µg/ml (Martinez-Gutierrez et al., 2013). In contrast, the amount of silver in α-Ag_2_WO_4_ at 7.81 µg/ml concentration is extremely lower (0.015 µg/ml; 0.033 µmol/ml) when compared with those silver nanoparticle solutions (Chávez et al., 2018) and still was able to reduce *C. albicans* by 1.5 log_10_. It is well known that silver could be toxic, not only to microorganisms but also to mammalian cells, causing DNA damage and even cell death. The range of concentration reported to be toxic to mammalian cells is between 10 and 100 µg/ml (Chernousova and Epple, 2013), far higher than those used in the present study. Also, silver size influences its toxicity, so that, at the same silver concentration, smaller silver particles are more toxic than larger ones for they can easily be taken up by cells and interact with nuclei acids and cell membranes (Chernousova and Epple, 2013). Previous studies have shown that α-AgVO_3_, α-Ag_2_WO_4_, and β-Ag_2_MoO_4_ are capable of producing ROS from the interaction between semiconductors (V, W, and Mo) with water and oxygen present in the medium (Fabbro et al., 2016; de Oliveira et al., 2017; Foggi et al., 2017a; Foggi et al., 2017b; Assis et al., 2019). This has been shown to be the principal mechanism involved in their antimicrobial activity. Through photoluminescence analysis, it has been observed that α-AgVO_3_, α-Ag_2_WO_4_, and β-Ag_2_MoO_4_ emit spectra in the blue and red regions, which are related to energy defects generated by microcrystals. These defects are responsible for promoting distortions in the molecules of microcrystals, generating OH* and •O_2_H*, and consequently, causing the death of microorganisms by oxidative stress (Fabbro et al., 2016; de Oliveira et al., 2017; Foggi et al., 2017a; Foggi et al., 2017b; Assis et al., 2019).

Morphological analyses of the 3D coculture models also demonstrate that α-Ag_2_WO_4_ had antibiofilm activity similar to FCZ. Furthermore, it was observed that these two groups were capable of better preserving the epithelial cell layer and contain the infection in the epithelial layer. On the other hand, infected 3D coculture models challenged with α-AgVO_3_ and β-Ag_2_MoO_4_, as well as the control, showed the absence of the epithelial layer and a thicker biofilm of *C. albicans* (CW) reaching the collagen matrix layer. The destruction of epithelial cells observed in those groups is typical of invasion of *C. albicans* biofilms. Dias et al. (2018) demonstrated that *C. albicans* biofilm was able to cause great alterations in an *in vitro* tissue model. It was observed that *C. albicans* hyphae is responsible for tissue invasion and destruction, even serving as bridge to other microorganisms that are unable to penetrate host tissue, such as *S. aureus* (Dias et al., 2018). The cell damage was also proven by lactate dehydrogenase level, which was expressively increased in tissue models when *C. albicans* was present in biofilms (Dias et al., 2018). This capacity of *C. albicans* to attach and penetrate host tissue is related to hydrolytic enzymes, such as proteases and phospholipases, which are secreted by this microbial biofilm (Ingham et al., 2012; Zago et al., 2015). In the host, these enzymes play an important role in inflammatory and pathological responses during infection (Villar and Zhao, 2010).

Regarding cytokine production, α-Ag_2_WO_4_ promoted an increase in IL-6 and IL-8 production by noninfected 3D coculture models. As one of the mechanisms of action described for microcrystals is related to the generation of ROS (Fabbro et al., 2016; de Oliveira et al., 2017; Foggi et al., 2017a; Foggi et al., 2017b; Assis et al., 2019), the oxidative stress generated could activate inflammatory pathways, such as MAPK and NF-κB, and then release proinflammatory cytokines, such as IL-6 and IL-8. It is possible that among the microcrystals analyzed in this study, α-Ag_2_WO_4_ is more reactive and, therefore, results in greater generation of ROS, which consequently would activate the proinflammatory pathways releasing more IL-6 and IL-8 (Park and Park, 2009). However, the increased levels of IL-6 and IL-8 when 3D coculture models were exposed to α-Ag_2_WO_4_ were not different from those observed in infected 3D coculture models. As expected, *C. albicans* biofilm also increased the production of IL-6, IL-1β, and IL-8. *C. albicans* is known to modulate the cellular inflammatory response and stimulate the production of IL-6, IL-1β, and IL-8 (Dongari-Bagtzoglou and Kashleva, 2003; Gow and Hube, 2012; Qin et al., 2016; Richardson et al., 2019). The increase in these cytokines is important for fungal infection control, as IL-6 is responsible for the recruitment of neutrophils (Cheng et al., 2012); IL-1β is related to IL-17 production, important for defense against *C. albicans* (Cheng et al., 2012), and IL-8 may act by increasing the anticandidal activity (Szolnoky et al., 2001) and proliferation of keratinocytes, essential for maintaining epithelial integrity and barrier function (Rennekampff et al., 2000). Despite demonstrated benefits, some silver-based materials can cause immunotoxicity if used at higher dosages with high amounts of silver ions. Such materials should be used within a therapeutic window that allows minimizing biofilm growth without leading to adverse inflammatory consequences and/or cytotoxicity.

## Conclusion

Taking into account the observed cell responses and antifungal behavior of the microcrystals tested in the present work, α-Ag_2_WO_4_ seems to be a more promising biomaterial when compared with the others, having the ability to modulate the inflammatory response and minimize the infectious process via antifungal activity. Also, the results obtained in the present work showed, for the first time, a 3D coculture model involving three cell lines with an inflammatory component that allow to better comprehend the action of biomaterials on human infected tissue. This model was considered a reliable tool for the biocompatibility assessment of α-AgVO_3_, α-Ag_2_WO_4_, and β-Ag_2_MoO_4_, as well as in the investigation of the antifungal activity of these microcrystals against *C. albicans* biofilms.

## Data Availability

The raw data supporting the conclusions of this article will be made available by the authors, without undue reservation.
